# Real-Time Measurement of the Daily Total Locomotor Behavior in Calves Reared in an Intensive Management System for the Possible Application in Precision Livestock Farming

**DOI:** 10.3390/vetsci10010064

**Published:** 2023-01-16

**Authors:** Claudia Giannetto, Raul Delmar Cerutti, Maria Cristina Scaglione, Francesca Arfuso, Melissa Pennisi, Elisabetta Giudice, Giuseppe Piccione, Alessandro Zumbo

**Affiliations:** 1 Department of Veterinary Sciences, University of Messina, 98168 Messina, Italy; 2 Department of Veterinary Sciences, Universidad National del Litoral, Pellegrini 2750, Argentina

**Keywords:** total locomotor activity, newborn calves, livestock, daily rhythm

## Abstract

**Simple Summary:**

Intensive farm conditions have an impact on welfare and can reduce the locomotor behavior of calves. The Actiwatch-Mini^®^ (Cambridge Neurotechnology Ltd., Cambridg, UK), an actigraphy-based data logger was used to record the total locomotor behavior of 30-day-old calves, which showed diurnal daily rhythmicity. An individual daily rhythm of total locomotor behavior has also been found and varied in the same subject from day to day, and from subject to subject, and these variabilities need to be taken into account during the farm monitoring.

**Abstract:**

Housing confinement, adaptation to different light/dark conditions, and social deprivation could modify the amount of total locomotor behavior of calves recommended for their psychophysical health. Total locomotor behavior was recorded by means of an activity data logger every 5 min for 6 consecutive days. To do that eight clinically healthy 30-day-old Holstein calves living in calf boxes under natural photoperiod and environmental conditions were enrolled. ANOVA (analysis of variance) showed a statistical effect of the day of monitoring and animal. In the temporal distribution of the resting–activity frequency, it was observed that the calves presented periods of total locomotor behavior with the existence of two peaks, one between 06:00–07:00 and another between 17:00–18:00, which corresponds to time of food intake. In all animals, a diurnal daily rhythm of total locomotor behavior was observed during time of monitoring. Intrasubject and intersubject variabilities were statistically different in mesor, amplitude, and robustness of rhythm. In conclusion, the total locomotor behavior showed a diurnal daily rhythmicity in 30-day-old calves. The characteristics of rhythm were different from individual to individual and from day to day. The recorded intersubject variability must be taken in consideration during the monitoring of farm animals and justifies the application of the device to each animal, as precision livestock farming suggests.

## 1. Introduction

In farm animals, behavioral rhythm, such as the lying, standing, and/or walking behavior, covers a research field of considerable interest, being an integral part of the species’ ecological interaction and part of its evolutionary compliance. Animal behavior monitoring is used to assess the animals’ health and welfare status in livestock production. Numerical scoring systems are applied to systemic and quantitative records of animal behavior to identify states of malaise such as injury or lameness [1].

Animals’ locomotor behavior is essential to sustain life, including food searching, avoiding danger, etc. Behavioral strategies are different among the various species on the basis of each ecological niche demand, with a consequent difference in the amount and type of movement required to maximize the use of their habitat [2]. The animals’ welfare state is the basis of modern breeding management, in association with genetic selection, the type of management, and the type of feed provided. The modern breeding system involves keeping animals indoors, cubicle housing systems have been widespread in many countries [3]. The indoor habit allows for controlling climatic and environmental conditions including the feed supply, temperature, humidity, bedding, and regular health checks [4]. Stabled animals are confined to restricted spaces with a consequent reduction in their physiological behavior [5], with the consequent manifestation of frustration and stress in the short term [6]. In the livestock system, there is a more interesting point for maximum efficiency and productivity from farm animals in order to ensure better economic profit. Intensive breeding leads to a stressful lifestyle with restriction of physical behaviors and, subsequently a disruption of the circadian rhythm, essential for the growth performance and well-being of animals [7]. Endogenous rhythms are correlated with environmental changes, and synchronized by them, although they are not necessarily a direct response to them. The locomotor behavior of all free-ranging animals is divided into periods of rest and activity, and they exhibit striking activity patterns [8].

Therefore, the effect of housing and farm management can be evaluated by monitoring animal behavior as an index of health status. [9]. In some cases, animals are confined within limited schedules and a narrow range of normal physiological activity, this affects features and amount of total locomotor activity [5]. Considering that changes in patterns and/or the daily rhythm of activity are often the first sign of disturbance, the detailed knowledge of behavioral consistencies can bring the best planning of daily management practices and help improve the planning of breeding facilities [10].

In a large number of mammal species, total locomotor activity daily rhythms have been documented. Cows have been classified among the diurnal animals having a stable total locomotor activity daily rhythm characterized by high robustness and high diurnality index [11]. It has been observed that the management conditions highly influenced the robustness of rhythm in this species. In particular, when animals have been housed under an artificial photoperiod and indoor ambient temperature a robustness of rhythm of about 51.0% has been observed [12]; the monitoring of total locomotor activity in cows housed in stable conditions showed robustness of rhythms of about 17.0% [13]. Additionally, housing conditions have been observed to influence the total locomotor activity daily rhythm reducing the amplitude value in cows living in stable respect to the free-ranging paddock animals [14]. The time used for rest depends on the time not used for other activities. In particular, in extensive farms, rest time is linked to feeding behavior, influenced by the hours of light and climatic conditions. In intensive farming, eating behavior is influenced by the availability of food controlled by humans, which therefore influences the period of rest [8]. The analysis of the lying, standing, and walking behavior is of great interest in livestock. Changes in activity and resting have been demonstrated to be related to a decrease in livestock productivity [15]. Cows need to lie from 8 to 15 h per day during which they ruminate. In young animals, a reduction in lying has been reported as a consequence of an increase in playful behavior [16].

Modern farm management (including precision livestock farming) provides for the monitoring of each individual animal through the application of monitoring devices. Sensor technologies open the possibility to analyze total locomotor behavior continuously improving management and animal welfare. Monitoring individual behavioral changes is key to exploiting the potential of individualized behavioral monitoring The purpose of the study was to evaluate the intra-subject and intersubject variability of the total locomotor behavior daily rhythm in 30 days old calves living in an intensive system.

## 2. Materials and Methods

**Figure 1:**
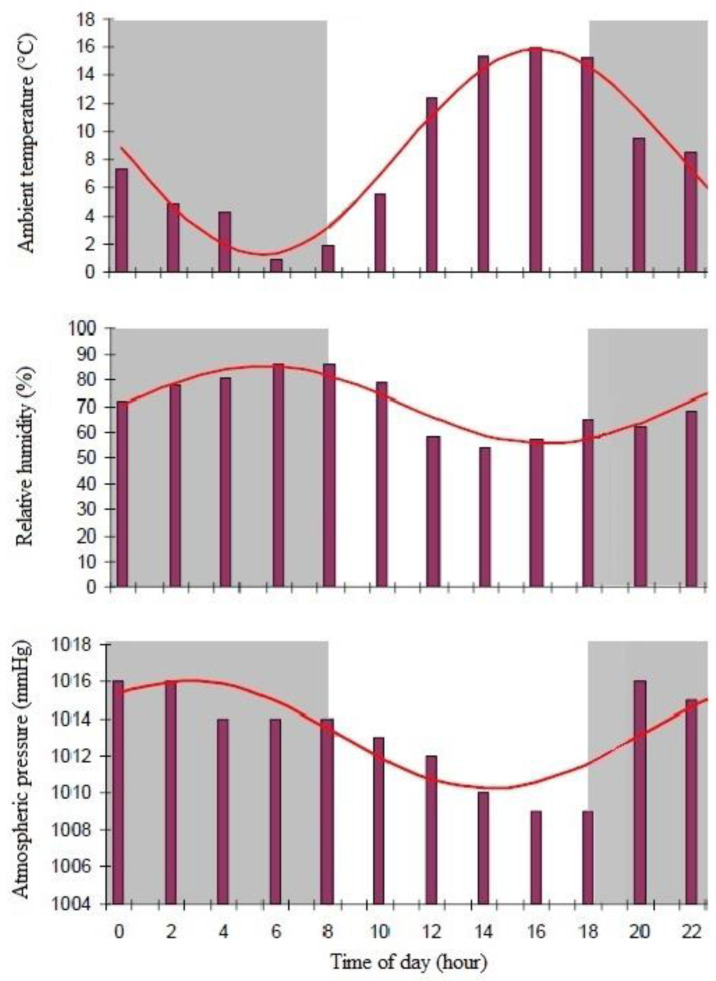
Rhythmic patterns of climatic variables during the trial of Holstein calves at 30 days of life subjected to natural conditions with periods of light–dark cycle 10L:14D. (Cont.) Y = A_0_ + A_1_ × Cos (w × t + Φ).

This study was carried out in a commercial milk production farm located in Villa Trinidad (Santa Fe, latitude 30°13′17.12″ and longitude 60°53′38.5″) and 96 m above sea level. The calves were randomly selected from a commercial-intensive dairy farm that receives veterinary assistance and technical advice on nutritional aspects. Eight female Holstein newborn calves (30 days old), clinically healthy, of similar weight (between 51 and 56 kg) housed in calf boxes equipped with a perforate rubber floor, under natural light/dark cycle (10L:14D) were enrolled. During the experimental period, the information on ambient temperature, atmospheric pressure, and relative humidity was collected from an automatic weather station located near the place where the experimental protocol was carried out (Figure 1). The calves enter the system at 7 days of life, once the animals arrive at the farm, they were placed in individual calf boxes for 5 weeks. During this period, the weight gain achieved is 400 gr./day on average.

Calves were fed with four liters of milk daily two times a day (06:30 and 16:30). Whole, non-GMO corn (*Zea mays*) kernel from plantation-in-place harvest used as crop rotation, alfalfa hay (Medicago sativa), obtained by harvesting surplus for deferred use, and water was available *ad libitum*.

The protocol of this study was reviewed and approved in accordance with the standards recommended by the Guide for the Care and Use of Laboratory Animals and Directive 2010/63/EU.

### 2.1. Locomotor Activity Recording

Total locomotor behavior including various conscious and unconscious movements due to feeding, drinking, grooming, and walking [17] was recorded in each animal for 6 consecutive days, with a sampling interval of 5 min. To record activity, the animals were equipped with Actiwatch-Mini^®^ (Cambridge Neurotechnology Ltd., Cambridge, UK), an actigraphy-based data logger, recording a digitally integrated measure of motor activity, placed on collars that did not disturb the animals’ behavior [18,19]. This activity acquisition system is based on miniaturized accelerometer technologies, currently used for human activity monitoring but also tested for activity monitoring in small non-human mammals [20,21]. and validated for automatic 24 h recording of activity in dairy cows [22]. Actiwatch-Mini utilizes a piezo-electric accelerometer that is set up to record the integration of intensity, amount, and duration of movement in all directions. This type of sensor integrates the degree and speed of motion and produces an electrical current that varies in magnitude. An increased degree of speed and motion produces an increase in voltage. The corresponding voltage produced is converted and stored as an activity count in the memory unit of the Actiwatch-Mini. Activity count is a generic term used to denote the amplitude of the signal produced by the accelerometer in the Actiwatch. The number of counts is proportional to the intensity of the movement. The piezo-electric accelerometer converts the voltage of the intensity, amount, and duration of behavior in activity count. The Actiwatch unit records all movement over 0.05 g and the maximum frequency is 32 Hz.

### 2.2. Statistical Analysis

Factorial variance analysis of variance (ANOVA) was applied to determine statistical differences in total locomotor behavior between the six days of monitoring in the same subject (intersubject variability) and between the eight subjects (intrasubject variability).

The recorded data were subjected to time series analysis. The existence of the periodic form and its characterization was carried out following the harmonic analysis or Fourier development. The statistical analysis was carried out using the SAS^®^ NLIN nonlinear regression model, with the Gauss–Newton methodology to obtain the first term of the Fourier series. The model for fitting data to a cosine function was as follows:

y_i_ = A_0_ + A_1_ × Cos (w × t + Φ)

(y_i_: = is the value of the i-th point of the data series of N points; A_0_ = mesor; A_1_ = amplitude; w = angular frequency; t = time expressed in hours; Φ = acrophase).

The adjusted regression coefficient (R) was used as the model adjustment parameter, which was determined as the square root of the ratio between the variance explained by the cosine model divided by the mean squared error.

For the construction of the polar graph, the center of each ellipse in parametric coordinates where:

X_0_ = A_1_ × sen Φ and Y^0^ = A_1_ × cos Φ

The points belonging to the ellipses that cover the confidence interval of the 95% were represented in parametric coordinates according to:

x = x_0_ + ESΦ/24 × A_1_ × cost × cos Φ − ES/24 × A_1_ − sent × sen Φ

y = y_0_ + ESΦ/24 × A_1_ × cost × sen Φ − ES/24 × A_1_ − sent × cos Φ

(y_i_: = is the value of the i-th point of the data series of N points; A_0_ = mesor; A_1_ = amplitude; w = angular frequency; t = time expressed in hours; Φ = acrophase; ES A_1_ = Standard error of the amplitude corresponding to a 95% confidence interval; ES Φ = Standard error of acrophase corresponding to a confidence interval of 95% and t = time expressed in hours).

The single cosinor procedure was applied to each subject time series so as to describe the periodic phenomenon analytically, by characterizing the main rhythmic parameters according to the single cosinor procedure [23]. Four rhythmic parameters were determined: mesor (mean level), amplitude (half the range of oscillation), acrophase (the time at which the peak of a rhythm occurs), and robustness (strength of rhythm). The mean level of each rhythm was computed as the arithmetic mean of all values in the data set, and the amplitude of a rhythm was calculated as half the range of oscillation, which in its turn was computed as the difference between peak and trough. Rhythm robustness was computed as a percentage of the maximal score attained by the chi-square periodogram statistic for ideal data sets of comparable size and 24 h periodicity [24]. Intrasubject and intersubject variabilities in the amount of the found circadian parameters (mesor, amplitude, acrophase, and robustness) were computed as the standard deviations of the means. The standard deviations of the mean of the eight calves across six days were used as the measure of intrasubject variability. Likewise, the standard deviations of the means for six days across the eight subjects were used as the measure of intersubject variability.

ANOVA was applied to determine any statistically significant effect of intersubject versus intrasubject variabilities on the rhythmic parameters. Bonferroni’s test was applied for post hoc comparisons. *p* value < 0.05 was considered statistically significant. The data were analyzed using the software STATISTICA 8 (Stat Soft Inc., Tulsa, OK, USA).

## 3. Results

The application of factorial ANOVA on the total locomotor behavior data recorded every 5 min showed a statistical effect of the day of monitoring (*p* < 0.0001) and a statistical effect of animal (*p* < 0.0001).

In order to analyze the rhythms of resting activity and establish whether fit at a given rate, a cosine fit was applied to the data recorded in 30-day-old Holstein calves for six days.

**Figure 2:**
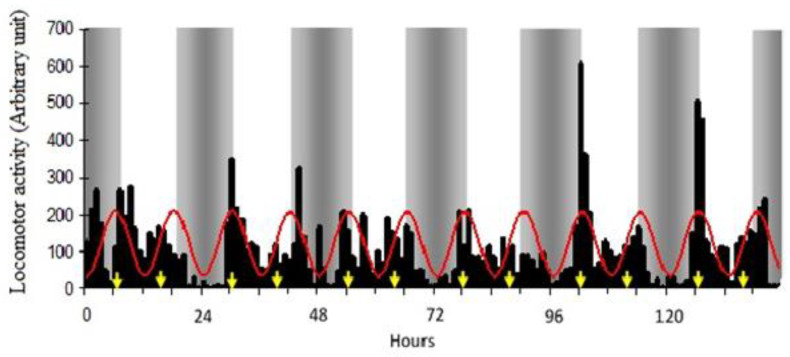
Rhythms of the resting activity of Holstein calves (bars) and curve obtained by applying the cosenoidal adjustment model to the variable under study (continuous line). The gray area indicates hours of darkness; yellow arrow indicates the time of food intake.

**Figure 3:**
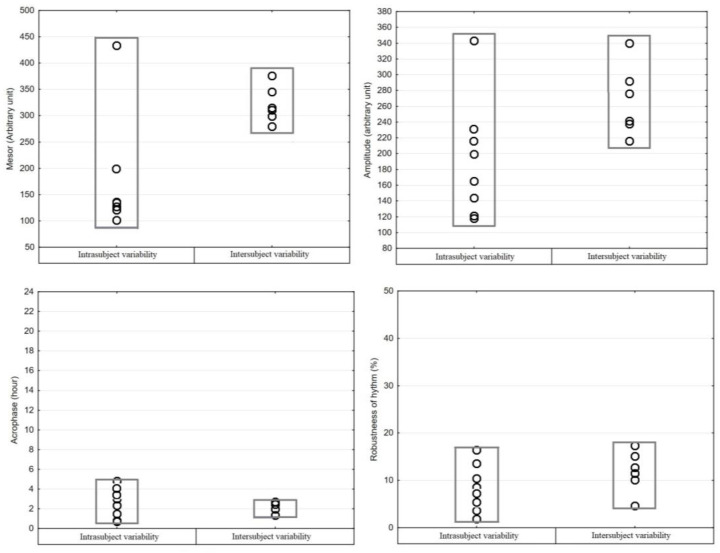
Results of the analysis of intra- and intersubject variability of the circadian parameters of the total locomotor activity rhythm recorded in eight calves for six consecutive days.

The graphical representation of the mean values of resting activity together with the cosine curves is shown in Figure 2. The value of the coefficient of correlation (R = 0.63) shows the adequate adjustment achieved by means of the nonlinear regression model, presenting an ultradian rhythm with a period of 12:02 h. In the temporal distribution of the resting activity frequency, it was observed that the calves presented periods of locomotor activity with the existence of two peaks, one between 06:00 and 07:00 and another between 17:00 and 18:00, which corresponds to the time of food intake. The application of the single cosinor method showed a daily rhythm of the total locomotor behavior in all groups, on all days of monitoring (Table 1). Intrasubject and intersubject variabilities were statistically different in mesor values (*p* < 0.02), amplitude (*p* < 0.05), and robustness of rhythm (*p* < 0.01). Acrophase (*p* = 0.86) did not show differences in intra and intersubject variability (Figure 3).

## 4. Discussion

Although numerous works have been carried out on bovine ethology [25,26], less attention has been focused on the study of total locomotor behavior rhythms. Our results showed that the activity was observed to a greater extent during light phases. Activity increased gradually during the hours preceding the light phase, with an abrupt rise at sunrise (08:00 a.m.). Thirty days after birth is the moment of life in which the postnatal phase ends, at this stage, the metabolic maturation of the animal is completed, in association with the maturation of the circadian clock, guaranteeing the adaptation to the extrauterine environment [27,28]. Calves showed a diurnal daily rhythm of total locomotor behavior, as previously observed in adult cows both during the milking and dry periods in which the photic stimuli have been considered the cause of the main activity recorded during the light phase with respect to the dark phase [8]. In addition, a relationship between activity and feeding presents the same behavior as for the stimulus of light with a gradual anticipatory increase and marked activity at the time of food supply. This means that aside from the light–dark cycle, food acquired relevance as a synchronizer of biological rhythms, presenting the resting activity as a bimodal curve, with a light–food synchronized component and another component associated only with food, which suggests the expression of two decoupled clocks. The restricted daily access to food has been demonstrated to act as a zeitgeber for anticipatory free-running rhythms of locomotor activity in rats [29], sheep [30], and goats [31] in which photic and non-photic stimuli entrained the daily rhythm of total locomotor activity.

Differences in the daily distribution of activities were observed both between species and between individuals belonging to the same species [32]. Differences in the activity recorded every 5 min were observed on the various days of monitoring in the same subject and among the different subjects on the same day of monitoring. The presence of different activity peaks between different subjects has been attributed to an individual response to noxae, but it is interesting to underline as the same animal subjected to the same daily routine reacted in a different way one day from another. The analysis of intrasubject and intersubject variability also identified an individual daily rhythm of total locomotor behavior that varied in the same subject from day to day, and from subject to subject. In this phenomenon, it is interesting to underline the stability of acrophase that did not change among subjects and among the different days of monitoring. Probably the cause of the changes in the mesor and amplitude of the daily rhythm of total locomotor behavior in 30-day-old calves living in calf boxes is the low robustness of the rhythm recorded. The low robustness of the rhythm has been considered to be responsible for the weakness of the locomotor activity daily rhythm [11].

## 5. Conclusions

Detailed knowledge of total locomotor activity consistencies may be to better scheduling of daily management practices and help to improve the design of facilities for livestock, also considering that the ecological niche of each particular animal species demands a different behavioral strategy. In 30-day-old calves, the total locomotor behavior showed diurnal daily rhythmicity. The characteristics of rhythm were different from individual to individual and from day to day. These findings have not been observed before, therefore it is necessary to verify if the recorded intrasubject variability was due to incomplete maturation of the circadian system involved in the control of total locomotor behavior, or to the farming management to which these animals were subjected. Little is known about locomotor activity in calves, further studies are needed to understand how the differences respect animals that are free to roam in a larger space with animal-to-animal contact may influence the locomotor behavior in the first 30 days of life. The recorded intersubject variability must be taken into consideration during the monitoring of farm animals and justifies the application of the device to each animal, as precision livestock farming suggests.

## Figures and Tables

**Table 1:** Circadian rhythm parameters (mesor, amplitude, acrophase, and robustness of rhythm) recorded during six days of monitoring in eight Holstein calves of 30 days of life, expressed as mean ± sd in their conventional unit.

	Mesor (Arbitrary Unit)	Amplitude (Arbitrary Unit)	Acrophase (hh:mm)	Robustness (%)
Day 1	1149.10 ± 134.94	376.59 ± 199.50	13:45 ± 45 min	23.88 ± 10.39
Day 2	1184.03 ± 120.63	739.17 ± 165.22	13:35 ± 40 min	25.23 ± 13.56
Day 3	1022.05 ± 135.80	466.87 ± 143.67	13:45 ± 3 h 01 min	8.58 ± 3.59
Day 4	843.25 ± 127.25	273.64 ± 118.11	13:50 ± 2 h 20 min	6.13 ± 1.86
Day 5	516.23 ± 101.71	254.97 ± 121.14	11:10 ± 1 h 30 min	10.25 ± 8.53
Day 6	721.79 ± 433.64	393.96 ± 343.06	12:15 ± 4 h 45 min	17.96 ± 16.40

## Data Availability

The data that support the findings of this study are available from the corresponding author upon reasonable request.
